# The Prognostic Impact of Histopathological Variants in Patients with Advanced Urothelial Carcinoma

**DOI:** 10.1371/journal.pone.0129268

**Published:** 2015-06-26

**Authors:** Meng-Che Hsieh, Ming-Tse Sung, Po-Hui Chiang, Cheng-Hua Huang, Yeh Tang, Yu-Li Su

**Affiliations:** 1 Division of Hematology-Oncology, Department of Internal Medicine, Kaohsiung Chang Gung Memorial Hospital, Kaohsiung, Taiwan; 2 Department of Pathology, Kaohsiung Chang Gung Memorial Hospital and Chang Gung University College of Medicine, Kaohsiung, Taiwan; 3 Department of Urology, Kaohsiung Chang Gung Memorial Hospital and Chang Gung University College of Medicine, Kaohsiung, Taiwan; 4 Division of Hematology-Oncology, Department of Internal Medicine, E-Da Hospital, I-Shou University, Kaohsiung, Taiwan; University of Central Florida

## Abstract

**Purpose:**

This study investigated the prognostic role of histopathological variants in patients with advanced urothelial carcinoma (UC) who were treated with systemic chemotherapy.

**Materials and Methods:**

We conducted a retrospective analysis of patients with unresectable and/or metastatic UC who underwent systemic chemotherapy between January 1997 and December 2013 in Kaohsiung Chang Gung Memorial Hospital. Histopathological types were categorized as pure UC (PUC) and variants of UC (VUC). The overall survival (OS) and progression-free survival (PFS) were calculated using Kaplan–Meier analyses and Cox proportional regression models.

**Results:**

A total of 206 patients were enrolled; 53 of the patients (25.7%) had histopathological variants. The most common variant was squamous differentiation (68%). Compared with patients with PUC, patients with VUC significantly exhibited upper urinary tract origin (75% vs 52%, *P* = .008), chronic renal insufficiency (40% vs 23%, *P* = .03), and carboplatin-based chemotherapy (28% vs 10%, *P* = .003). According to univariate analysis, the median OS for PUC patients was significantly higher than that for VUC patients (15.9 vs 11.3 months, *P* = .007). The median PFS for patients who received first-line chemotherapy was 6.1 and 3.8 months for PUC patients and VUC patients, respectively (*P* = .004). Multivariate analysis revealed that VUC (hazard ratio [HR] 1.67, 95% confidence interval [CI] 1.16–2.40, *P* = .006), an age ≤ 60 years (HR 0.70, 95% CI 0.49–0.99, *P* = .045) and presence of visceral metastasis (HR 1.54, 95% CI 1.11–2.13, *P* = .009) were independent factors facilitating OS prediction.

**Conclusions:**

The presence of histopathological variants indicates poor survival outcomes in patients with metastatic UC. Accordingly, VUC should be integrated into and considered an independent factor in a predictive model of survival.

## Introduction

In the United States, genitourinary tract cancer was the fourth and eighth most common cancer in men and women, respectively [1] The major histopathological type of genitourinary tract cancer, including that of the upper urinary tract and the bladder, is urothelial carcinoma (UC). Previous studies have shown that UC has the propensity for divergent differentiation into various histologic subtypes [2], with an incidence of 7%–81% [3, 4] The most common histopathological variant is squamous differentiation, followed by glandular differentiation [5, 6]. Furthermore, several studies have revealed that compared with pure UC (PUC), variants of UC (VUC) harbored aggressive biological features, such as advanced stage, higher grade, more tumor necrosis, tumor multifocality, lymphovascular invasion and lymph node metastasis [7, 8] Recent evidence suggested that VUC can be used to predict survival, regardless of whether patients have upper urinary tract UC (UUTUC) or UC of the bladder (UCB). In studies investigating radical cystectomy or nephroureterectomy series, approximately 25% of patients with UCB or UUTUC had histopathological variants, and the oncologic outcomes were significantly poorer than those of patients with PUC [8, 9]. Regarding histopathological variants, UC patients with squamous, glandular, micropapillary, and nested differentiation had similar survival outcomes [9–13], whereas patients with plasmacytoid differentiation had the worst prognosis [14, 15]. Notably, all of the aforementioned studies were mainly focused on patients treated with radical surgery. However, the prognostic role of histopathological variants in patients with metastatic UC has never been investigated. Because no conclusive study exists at present, the purpose of the current study was to investigate the impact of histopathological variants on the survival of patients with metastatic VUC.

## Materials and Methods

### Study population

**Fig 1:**
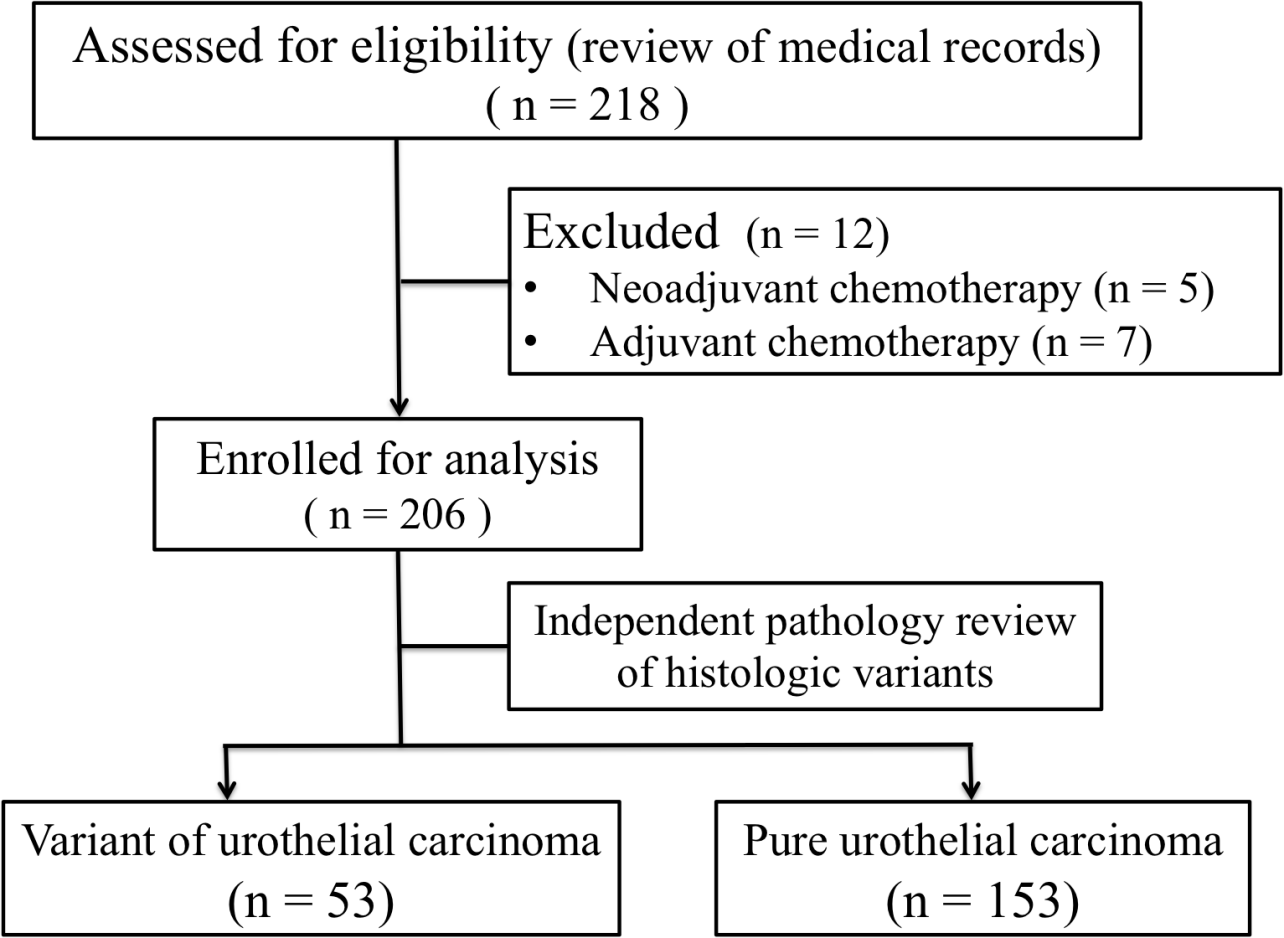
A Consort diagram.

We conducted a retrospective review of a database comprising information about patients who received systemic chemotherapy for metastatic UC at Kaohsiung Chang Gung Memorial Hospital between January 1997 and December 2013. Patients with unresectable and/or metastatic UCB or UUTUC were enrolled and stratified according to histopathological variants labeled as VUC and PUC, respectively (Fig 1). Patient characteristics were assessed and recorded in the database, which included age, sex, Eastern Cooperative Oncology Group (ECOG) performance status, tumor origin, histopathological type, baseline renal function, sites of metastases, and chemotherapy regimens. Most of the pathologic slides were viewed and reported by a dedicated urologic pathologist, and definitions of variants were interpreted according to the World Health Organization (WHO) classification [16]. In addition, we performed review of slides for VUC patients by an independent pathologist who was masked for patient’s information and original pathologic diagnosis. Palliative radiotherapy or surgery for symptoms control was allowed. The chemotherapy regimen was determined individually by the treating physician. Patients without pathologic proof, history of systemic chemotherapy, or nonplatinum-based first-line chemotherapy were excluded. Oncologic evaluation consisted of physical examination and imaging tools including chest roentgenogram, abdominal ultrasound, computed tomography (CT), and/or bone scan in a frequent follow-up period. Disease progression was defined as distant metastasis or local failure in the operative site or regional lymph nodes.

### Outcome analysis

The primary objective of this study was to assess overall survival (OS), which we defined as the time from metastatic disease diagnosis to the date of death. The secondary objectives were to evaluate the progression-free survival (PFS) and objective response rate (ORR) of patients who received first-line platinum-based chemotherapy. The PFS was calculated from the date of metastasis to the date that the disease worsened or death. The ORR was defined as the percentage of complete response (CR) and partial response (PR) according to RECIST (Version 1.1). Patients without disease progression or death were censored at the date of their last follow-up for survival analysis.

### Statistical analysis

All demographic characteristics of patients with PUC and VUC were compared using the Pearson χ^2^ test. Kaplan–Meier curves and a log-rank test were used to estimate the PFS and OS. Cox proportional hazards models were used to assess the PFS and OS in a multivariate analysis. All statistical tests were 2-sided, and *P* values < .05 were considered statistically significant. All statistical analyses were performed using IBM SPSS software, Version 21 (SPSS, Chicago, Illinois, USA).

### Ethics statement

The retrospective study was approved by the Chang Gung Medical Foundation Institutional Review Board. Because this was a retrospective study involving no human tissue samples, the Institutional Review Board waived the requirement for informed consent from patients or their next of kin.

## Results

### Baseline characteristics

A total of 206 patients were enrolled; their median age at diagnosis was 63 years (22–84 y). There were 27 patients (13.1%) diagnosed by core-needle biopsy and 37 patients (18.0%) received TURBT. The others (68.9%) underwent radical cystectomy or nephroureterectomy. Patients with PUC and VUC constituted 74% (n = 153) and 26% (n = 53) of study cohort. As shown in Table 1, no significant differences were observed for most of the patient characteristics between the PUC and VUC groups, except for the location of the primary tumor, baseline renal function, and first-line chemotherapy. Approximately half of the PUCs were primarily located in the urinary bladder (47%), whereas the VUCs mostly occurred in the UUT (75%). Moreover, more histopathological variants were observed in metastatic UUTUC (34%) than in metastatic UCB (14%). Patients with VUC had a higher proportion of chronic renal insufficiency (defined as creatinine clearance rate < 60 mL/min) than did patients with PUC (40% vs 23%, respectively, *P* = .03). The most common metastatic site in both groups was lymph node (PUC, 74% vs VUC, 74%, *P* = 1.0), and the proportions of visceral metastases or the number of metastatic sites differed nonsignificantly between the 2 groups. Patients with PUC tended to receive more first-line cisplatin-based chemotherapy than did patients with VUC (90% vs 72% of PUC and VUC, *P* = .003). When the disease progressed after first-line chemotherapy, no significant differences were noted between patients with PUC and VUC receiving subsequent second-line chemotherapy.

**Table 1:** Clinical characteristics of 206 patients with advanced urothelial carcinoma.

	PUC		VUC		*p* value
	*N* = 153 (%)		*N* = 53 (%)		
Gender					0.31
Male	99	65%	39	74%	
Female	54	35%	14	26%	
Age (years)					0.19
≤ 60	66	43%	17	32%	
> 60	87	57%	36	68%	
ECOG Performance status					0.97
0–1	118	77%	41	77%	
≥ 2	35	23%	12	23%	
Renal function (mL/min)					0.03
CCr ≥ 60	117	77%	32	60%	
CCr < 60	36	23%	21	40%	
Location of primary tumor					0.008
Upper urinary tract	79	52%	40	75%	
Bladder	72	47%	12	23%	
Both	2	1%	1	2%	
Time to disease metastasis					0.43
At diagnosis	68	44%	27	51%	
After radical surgery	85	56%	26	49%	
Metastatic sites					
Lymph node	113	74%	39	74%	1.0
Visceral organs	42	28%	19	36%	0.3
Number of metastatic sites					0.74
1	95	62%	31	59%	
≥ 2	58	38%	22	41%	
1^st^ line chemotherapy					0.003
Cisplatin-based	137	90%	38	72%	
Carboplatin-based	16	10%	15	28%	
≥ 2^nd^ line chemotherapy					0.87
No	98	64%	35	66%	
Yes	55	36%	18	34%	

Abbreviations: PUC, pure urothelial carcinoma; VUC, variants of urothelial carcinoma; CCr, clearance of creatinine; ECOG, Eastern Cooperative Oncology Group

### Distribution of histopathological variants, treatment response, and survival outcomes

**Fig 2:**
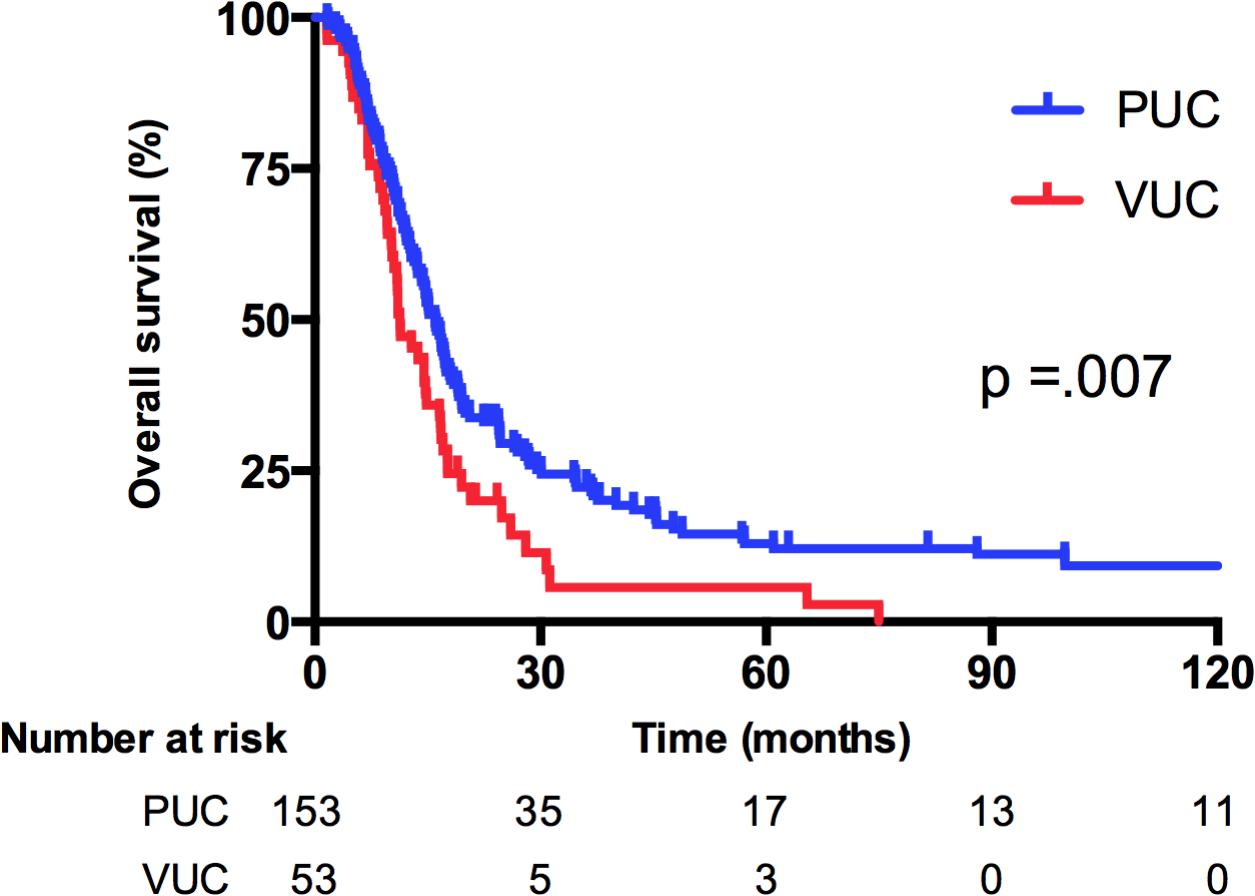
Overall survival (OS) stratified by variants of urothelial carcinoma (VUC) or pure urothelial carcinoma (PUC).

**Fig 3:**
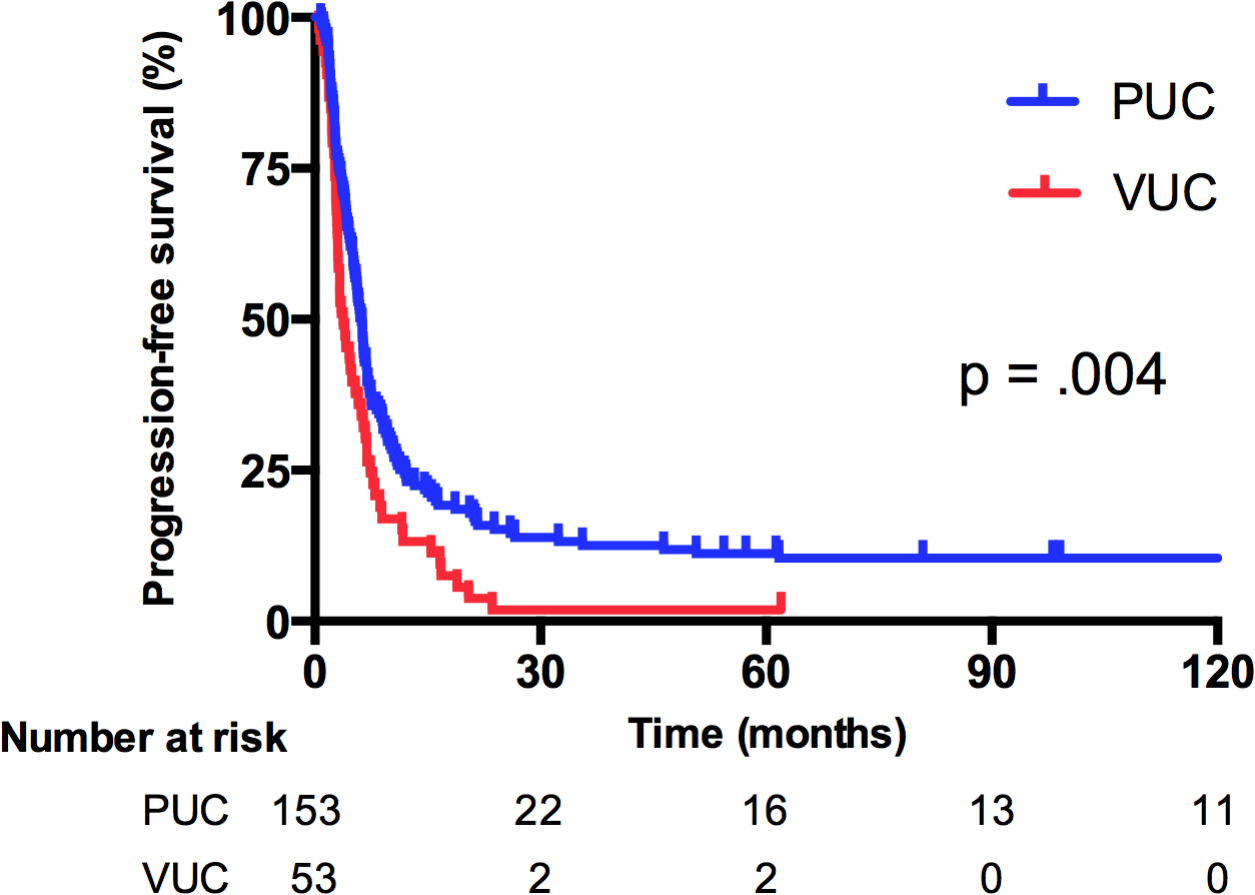
Progression-free survival (PFS) stratified by variants of urothelial carcinoma (VUC) or pure urothelial carcinoma (PUC).

Table 2 details the distribution of histopathological variants. Among the 53 patients with VUC, squamous differentiation (including one pure squamous cell carcinoma) was the most common histopathological subtype (68%), followed by micropapillary (11%), glandular (7%), and sarcomatoid differentiation (4%). Notably, 10% of the patients with VUC had synchronous 2 histopathological variants within one tissue specimen. Every patient enrolled in the study received systemic platinum-based chemotherapy. Gemcitabine and cisplatin (GC) was the most commonly received chemotherapy regimen in both groups (PUC, 45.1%; VUC, 47.2%), and a higher proportion of patients with PUC underwent methotrexate/vinblastine/doxorubicin/cisplatin (MVAC) chemotherapy than did patients with VUC (44.4% vs 24.5%, respectively, *P* = .002). For patients unfit for cisplatin, gemcitabine and carboplatin was the most commonly used regimen. Regarding the treatment response, 25 patients with PUC (16.3%) and 1 patient with VUC (1.9%) exhibited a CR after first-line chemotherapy. The proportion of a PR was similar between groups (PUC, 45.0% vs VUC, 43.4%). The ORR to first-line chemotherapy for PUC patients was 61.4%, indicating a marginal trend of significance (*P* = .053) when compared with the ORR of VUC patients (45.3%). During a median follow-up period of 134.5 months (range 1.55–183.7), 86% and 92% of patients with PUC and VUC died, respectively. Tumor progression contributed to the major cause of death in 97% of the PUC and 98% of the VUC patients. In Fig 2, the results of the Kaplan–Meier analyses revealed that patients with PUC had a significantly more favorable survival than did patients with VUC. The median OS was 15.9 versus 11.3 months (*P* = .007) for patients with PUC and VUC, respectively. Fig 3 illustrates the PFS. The median PFS for first-line platinum-based chemotherapy was 6.1 versus 3.8 months (*P* = .004) for patients with PUC and VUC, respectively.

**Table 2:** Distribution of histopathological variants.

Variants of urothelial carcinoma	N	%
Squamous differentiation	36	68%
Micropapillary differentiation	6	11%
Glandular differentiation	4	7%
Sarcomatoid differentiation	2	4%
Mixed squamous and sarcomatoid differentiation	2	4%
Mixed squamous and glandular differentiation	1	2%
Mixed squamous and micropapillary differentiation	1	2%
Mixed squamous and plasmacytoid differentiation	1	2%

### Univariate and multivariate Cox regression analyses

After performing univariate survival analysis, we conducted multivariate analyses, including all significant factors and using Cox proportional hazards models, to determine the PFS and OS. The independent variables were patient age, ECOG performance status, baseline renal function, site of primary tumor, VUC, presence of visceral metastasis, and chemotherapy regimen (Table 3). Histopathological variants (*P* = .007), age (*P* = .003), baseline renal function (*P* = .011), and visceral metastasis (*P* = .009) were significantly associated with prognosis according to the univariate analyses. Among the patients with VUC, no survival difference between squamous differentiation and nonsquamous differentiation variants were observed (*P* = .64). The results of multivariate Cox regression analyses revealed that histopathological variant was an independent prognostic factor for PFS (HR 1.53, 95% CI 1.10–2.15, *P* = .013) and OS (HR 1.67, 95% CI 1.16–2.40, *P* = .006) after adjustment for all confounders. In addition, the baseline renal function was a strong predictor for PFS (HR 0.59, 95% CI 0.40–0.87, *P* = .008), and presence of visceral metastasis was another independent factor for PFS (HR 1.92, 95% CI 1.40–2.64, *P* < .0001) and OS (HR 1.54, 95% CI 1.11–2.13, *P* = .009).

**Table 3:** Univariate and multivariate analyses of PFS and OS.

Characteristics	PFS			OS		
	Univariate	Multivariate		Univariate	Multivariate	
	*p* value	HR (95% CI)	*p* value	*p* value	HR (95% CI)	*p* value
Age ≤ 60 vs. > 60	0.17	0.83 (0.60–1.16)	0.28	0.003	0.70 (0.49–0.99)	0.045
ECOG PS 0–1 vs. ≥ 2	0.70	1.00 (0.68–1.47)	0.99	0.16	0.86 (0.58–1.28)	0.46
CCr (mL/min) ≥ 60 vs. < 60	0.004	0.59 (0.40–0.87)	0.008	0.011	0.72 (0.49–1.08)	0.11
Primary site UUT vs. bladder	0.43	1.13 (0.83–1.52)	0.44	0.56	0.85 (0.62–1.17)	0.31
Histologic variant VUC vs. PUC	0.004	1.53 (1.10–2.15)	0.013	0.007	1.67 (1.16–2.40)	0.006
Visceral metastasis Yes vs. No	< 0.0001	1.92 (1.40–2.64)	< 0.0001	0.009	1.54 (1.11–2.13)	0.009
1^st^ line chemotherapy Cisplatin- vs. Carboplatin-based	0.44	1.48 (0.90–2.44)	0.12	0.24	1.33 (0.81–2.18)	0.26

Abbreviations: CCr, clearance of creatinine; CI, confidence interval; ECOG, Eastern Cooperative Oncology Group; HR, hazard ratio; OS, overall survival; PFS, progression-free survival; PS, performance status; PUC, pure urothelial carcinoma; UUT, upper urinary tract; VUC, variants of urothelial carcinoma

## Discussion

We conducted a retrospective study to evaluate the impact of histopathological variants on the survival of patients with metastatic UC. Our results indicated that VUC was significantly, independently, and negatively associated with OS and PFS after adjustment for confounding factors. To the best of our knowledge, this is the first study exploring the impact of histopathological variant on the survival of patients with metastatic UC. The results suggest that histopathological variants should be considered crucial prognostic factors.

Urothelial carcinoma, previously known as transitional cell carcinoma, is a major histopathological type of genitourinary tract malignancy. Previous studies have reported that approximately 18%–25% of patients have been treated with radical nephroureterectomy harbored histopathological variants [8, 9]. Squamous differentiation is the most common variant, followed by glandular and sarcomatoid differentiation [8]. In the present study, we determined that 25.7% of metastatic UC had features of variant histology, and the majority of VUC was squamous differentiation (68%). Our cohort contained a higher proportion of patients with UUTUC (58%) because Taiwan has a high prevalence of UUTUC, which accounts for as much as 30% of all UC [17]. We observed VUC patients had a higher percentage of cancer originating from the UUT, chronic renal insufficiency and received more first-line carboplatin-based chemotherapy compared with the PUC patients. The reason VUC tended to originate from the UUT instead of the urinary bladder remains unknown. In fact, in this study, the proportion of VUC originating from the UUT (34%) was similar to that reported from a previous large radical nephroureterectomy (RNU) series (24.2%) [8]. A recent study by UUTUC collaboration showed that the median renal function decreased 18.2% for patients receiving RNU [18]. Thus, when patients with UUTUC experienced tumor recurrence, more patients developed chronic renal insufficiency and were thereby unqualified to undergo cisplatin-based chemotherapy. A previous study revealed that cisplatin-based chemotherapy might enable more favorable OS than does carboplatin-based chemotherapy in patients with metastatic UC [19]. By contrast, Dogliotti et al [20] conducted a phase II study to compare gemcitabine/cisplatin and gemcitabine/carboplatin chemotherapy in UC. No significant differences were noted in the overall response rate, time to tumor progression, and OS between 2 treatment groups. The results of the present study revealed that cisplatin-based chemotherapy is not an independent prognostic factor for survival according to both univariate and multivariate analyses.

Numerous studies have investigated the prognostic role of histopathological variants on survival outcomes. Most of such studies have focused on patients after radical surgery rather than on patients with metastatic diseases. Rink et al [8] reported that histopathological variants of UUTUC had more aggressive biological behaviors such as advanced tumor stage, lymphovascular invasion, sessile architecture, tumor necrosis, and lymph node metastasis. VUC was significantly associated with reduced recurrence-free survival and cancer-specific survival in univariate analysis; however, VUC was nonsignificant when included in multivariate analyses [8]. Lee and the colleagues showed the survival impact of squamous and/or glandular differentiation was statistically significant in patients with UUTUC, but not in patients with bladder cancer [21]. Another contemporaneous study by Kim et al. who analyzed a total of 1,037 radical cystectomy patients showed a similar survival of squamous/glandular differentiation compared with that of PUC [9]. Recently, Mitra et al conducted a case-control study to compare the survival between patients with PUC and those with variant squamous/glandular differentiation after radical cystectomy. When they matched demographic factors including age, sex, and pathologic stage, no differences in survival outcomes between the 2 groups were observed [22]. However, regarding patients with metastatic diseases who require chemotherapy, little evidence is available to examine the survival impact of histopathological variants. Our results indicated that the metastatic VUC patients had a PFS decline of 2.3 months and an OS decline of 4.6 months compared with the metastatic PUC patients. After adjustment for all confounding factors, VUC remained an independent and negative predictive factor for PFS and OS. This finding should be emphasized because the prognostic role of histopathological variants in metastatic UC has never been documented. In a recent study, Galsky et al developed a predictive nomogram to estimate the OS of patients with metastatic UC who received cisplatin-based chemotherapy [23]. According to analyses of 399 patients in prospective phase II or III trials, Galsky et al identified 5 predictive factors, namely elevated white cell count, numbers of metastatic sites, ECOG performance status, site of primary tumor, and lymph node metastasis. However, histopathological variants were not considered. We suggest that histopathological variants should be recognized and incorporated into prognostic models.

Although investigators exert considerable effort and enthusiasm in exploring the role of histopathological variants, many questions remain to be answered and are attributed mainly to the lack of molecular profiles and the unknown mechanism of histopathological differentiation and underlying carcinogenesis. A study on cDNA-microarrays showed that both keratin-10 and caveolin-1 were abundant in UC with squamous differentiation [24]. Compared with conventional UCB, pure squamous cell carcinoma displayed a highly significant expression of epithelial markers such as CK5/6 and CK 5/14 in parallel with negative staining for CK20 and uroplakin III [25]. Alexander et al examined the role of human papillomavirus (HPV) in squamous cell carcinoma of the urinary bladder by investigating p16 immunohistochemical expression and HPV DNA in situ hybridization [26]. Although p16 was detected in one-third of the squamous variants, Alexander et al observed no detectable HPV DNA or protein in the squamous cell carcinoma or squamous differentiation of the urinary bladder. Recently, Choi et al classified muscle-invasive bladder cancer (MIBC) into 3 molecular subtypes: basal, luminal, and p53-like MIBCs. The basal tumors were histopathologically enriched with sarcomatoid and/or squamous features and associated with shorter OS and disease-specific survival [27]. In addition to presenting with the expression of epithelial cytokeratins, basal tumors also presented with mesenchymal biomarkers and were characterized by p63 activation. Choi et al concluded that squamous and/or sarcomatoid differentiation of UC may be classified into basal-type urothelial cancer, which is significantly characterized by aggressive behavior and poor survival. In the present study, we observed a 4.6 month decline in OS for patients with VUC, a result that is consistent with those in the aforementioned reports.

Our study has several limitations. First, the retrospective and nonrandomized study design may limit the generalizability of the results, limiting information pertaining to effective treatment schema, evaluation of therapeutic response, and detailed records of adverse effects. Because of the referral nature of our practice, we adjusted the intensity of chemotherapy according to the patient performance status, age, and comorbidities. Although the results were not obtained from a rigorous clinical trial, they reflect the “real world” situation and can be used by clinicians in daily practice. Second, the sample size was relatively small, limiting the statistical power. Finally, some patients (13.1%) were diagnosed with UC solely by using a small biopsy specimen, which represents only part of a tumor, limiting the accuracy of pathologic diagnosis and the extent of histologic variants, which may be independent prognostic factors for survival [28]. As we performed pathology review of VUC patients only, some patients with variant features might be missed in PUC group, especially for whom were diagnosed only on a small core biopsy.

## Conclusions

In patients with metastatic UC, VUC was significantly associated with poor outcomes of PFS and OS. A significantly higher number of patients with VUC tended to have UUT origin and impaired renal function and received carboplatin-based chemotherapy. The responses to first-line chemotherapy between patients with PUC and VUC were comparable. Additional studies are required to define the underlying molecular pathways of VUC and to determine a potential target for treatment in such cases.
